# Awareness and perception of invasive fungal diseases among the Nigerian population

**DOI:** 10.4102/jphia.v16i1.1323

**Published:** 2025-10-28

**Authors:** Olufunmilola Makanjuola, Ubong A. Udoh, Damilola Akinlawon, Folasade Ogunsola, Rita Oladele

**Affiliations:** 1 Department of Medical Microbiology and Parasitology, University of Ibadan, Ibadan, Nigeria; 2 Department of Medical Microbiology and Parasitology, University of Calabar, Calabar, Nigeria; 3 Department of Community Health and Primary Care, Lagos University Teaching Hospital, Lagos, Nigeria; 4 Department of Medical Microbiology and Parasitology, College of Medicine, University of Lagos, Lagos, Nigeria

**Keywords:** invasive fungal diseases, invasive fungal infections, awareness, Nigeria, mycoses

## Abstract

**Background:**

Invasive fungal diseases (IFDs) are a public health issue causing morbidity and mortality in millions annually, yet they remain under-recognised.

**Aim:**

To determine the awareness and perception of IFDs among Nigerians.

**Setting:**

Three states in Nigeria: Lagos, Oyo and Cross River.

**Methods:**

This was a cross-sectional study utilising multistage sampling to recruit participants who responded to an interviewer-administered questionnaire. Logistic regression was used to determine factors associated with awareness and perception of IFDs, and a *p*-value of < 0.05 was taken for statistical significance.

**Results:**

One thousand two hundred and forty one participants were recruited with a mean age of 37.1 ± 16.1 years. The highest percentage had tertiary education as the highest educational attainment 538 (43.4%) and monthly household income within the lowest range of less than 30 000.00 naira ($40.00) 320 (25.8%). Awareness of IFDs was low 240 (19.3%) among the participants. Most participants 820 (66.1%) also had poor perception of fungal diseases. Tertiary education level showed higher IFD awareness (*p* < 0.001) than lower educational levels. Higher educational level was also associated with better perception, with secondary (*p* = 0.049), tertiary (*p* < 0.001) and postgraduate (*p* < 0.001) participants showing significantly better perception compared to those without formal education.

**Conclusion:**

Awareness and perception of invasive fungal infections among the Nigerian populace were low. There is a need for public health awareness and education on IFDs in Nigeria.

**Contribution:**

This study provides baseline data and crucially highlights a need for increased public health campaigns to improve awareness of IFDs in Nigeria.

## Introduction

The global annual burden of fungal diseases, ranging from minor skin and mucous membrane infections to serious life-threatening systemic diseases, has been estimated at billions, with millions estimated to have severe infections.^1^ Ensuing mortalities from life-threatening fungal infections have also been reported to be over a million cases annually worldwide.^1^ Though the mortality because of fungal diseases is comparable to those from other diseases targeted in the sustainable development goals (SDG), such as tuberculosis and malaria, fungal diseases, however, are relatively neglected by the general public and health authorities in many countries across the world.^2^

Very little attention is focused on the control and management of fungal infections, and people in many communities are oblivious of the disease burden, progression and ramifications. A recent report by the Centers for Disease Control and Prevention (CDC), aimed at guiding public health educational efforts on invasive fungal diseases (IFDs) in America, showed a low awareness across all fungal diseases. This low awareness varied by disease from 4.1% for blastomycosis to 24.6% for candidiasis. More than two-thirds (68.9%) of respondents had never heard of any of the diseases.^3^

Despite these alarming statistics, research funding and public health policies continue to overlook fungal diseases, diverting more resources toward bacterial and viral infections, and today there are still significant gaps in fungal disease diagnosis, surveillance and management, particularly in low- and middle-income countries (LMICs).^2^ Many healthcare professionals are also unaware of the latest advancements in fungal diagnostics and treatment, further compounding the challenges in tackling these infections effectively.^3^

Accounting for over 16% of the global population, Africa is the second most populated continent in the world^4^ and has a significant proportion of underlying risk factors for serious fungal diseases such as human immunodeficiency virus (HIV), diabetes and tuberculosis (TB).^5,6,7,8,9^ Human immunodeficiency virus prevalence in Africa has not only added to the burden of tuberculosis but has also influenced the burden of cryptococcal infections and *Pneumocystis jirovecii* pneumonia.^5,9,10^ Immunosuppression caused by HIV weakens host defences, making this population highly vulnerable to opportunistic fungal infections.^6^ Sub-Saharan Africa currently has the highest global burden of cryptococcal meningitis.^10^ It had been reported by the CDC that across all ages, candidaemia rates were approximately twice as high among Black people compared with other races and ethnicities.^11^

Developing countries such as Nigeria are likely to suffer a more intense negative impact concerning morbidity and mortality from severe fungal diseases because they have a high population of at-risk groups and are more resource limited.^3,9,12^ Nigeria, as the seventh most populated country in the world and the most populated country in Africa,^4^ has been estimated to have one of the highest burdens (11.8%) of invasive fungal infections here in Africa.^9^ The country has the sixth highest burden of tuberculosis in the world^5^; this condition is the most significant risk factor for chronic pulmonary aspergillosis.^13^

Unfortunately, the burden of fungal diseases in LMICs like Nigeria is exacerbated by the lack of awareness and widespread surveillance systems, leading to underreporting from the populace and misdiagnosis from the healthcare front. In many healthcare facilities, fungal infections are often mistaken for bacterial infections, resulting in inappropriate antibiotic use and worsening antimicrobial resistance. Furthermore, inadequate access to antifungal medications because of high costs and limited supply chains further contributes to poor health outcomes in affected populations.^9^

Understanding the level of awareness and public perception regarding diseases of public health significance is essential for developing effective health communication and intervention strategies. Surveys that assess awareness and perception offer critical insights into prevailing knowledge gaps, misconceptions and community attitudes, thereby guiding the creation of targeted and culturally sensitive educational interventions. Evidence from successful public health campaigns – such as those addressing TB and HIV – demonstrates that well-informed, media-driven outreach can significantly elevate awareness, even in underserved or low-literacy populations.^14^ Building on this model, electronic and social media campaigns can be strategically deployed to raise the profile of IFDs, which have remained largely neglected despite their increasing burden. This survey was conducted by a fungal disease capacity-building and surveillance programme prior to any intervention, with the goal of determining the level of awareness and perception of IFDs among the general populace in Nigeria.

## Research methods and design

### Study design and sample size determination

This was an analytic cross-sectional study of the adult populace in three states in Nigeria, assessing the awareness and perception of IFDs. The minimum sample size for this study was determined using Cochran’s formula for cross-sectional studies. Utilising an estimated awareness of 31.1% from a similar study by the CDC,^3^ and making a 10% adjustment for non-response, the minimum sample size calculated was 367. However, to enhance the robustness of the findings, a total of 1241 participants were ultimately included in the study.

### Sampling methodology

Multistage sampling was used to recruit the participants in the study. Stage one involved the use of the stratified sampling method to determine the number of respondents to be proportionally selected in Lagos, Oyo and Cross River following the ratio of 55:29:16, respectively. This allocation was based on population estimates of the states,^15^ ensuring that the sample represented the diverse demographic structures of the regions. In the second stage, cluster sampling was applied. Each state was divided into three senatorial zones to ensure geographic coverage. Within each zone, three clusters were purposively selected to represent key public gathering locations – markets, schools and religious centres – resulting in nine clusters per state and 27 clusters in total across the three states.

At the third stage, consecutive sampling was used within each selected cluster. Trained field staff approached eligible adults present at these locations and invited them to participate in the study. Eligibility criteria included being 18 years or older and providing informed consent. Approximately 40–50 participants were targeted per cluster to meet the required sample size per state and accommodate potential non-response.

The choice of specific schools, markets and religious centres within each senatorial zone was informed by factors such as accessibility, safety and approvals from local authorities. Although the participant selection within clusters was not randomised because of logistical constraints, efforts were made to minimise selection bias by varying the timing and days of recruitment to capture a diverse cross section of the population.

### Data collection method and instrument

Data were collected over 8 weeks from February 2022 to March 2022, using an interviewer-administered questionnaire adapted from a similar study done by the CDC.^3^ The study questionnaire explored each respondent’s information in three sections to capture comprehensive data (see Appendix 1). The first section included socio-demographic data consisting of the participants’ age, sex, marital status, highest educational level attained, average monthly income of household, history of visits to see the doctor in the past 12 months, history of chronic illness and number of chronic medications utilised by participants. The second section comprised awareness of IFDs, where participants’ awareness was assessed through a direct question that listed specific IFDs – invasive candidiasis, cryptococcosis, aspergillosis, mucormycosis and histoplasmosis – where respondents indicated whether they had ever heard of each infection. The third section comprised the perception of IFDs, which consisted of ten questions arranged in a three-point Likert scale format, where participants could choose between a correct response, an uncertain response or an incorrect response.

The data instrument was pre-tested on the University of Lagos staff and students (undergraduate and postgraduate), where fifty questionnaires were shared randomly to ascertain clarity and validity of the questionnaire. The pre-test aimed to identify and address any potential ambiguities in the questions and verify the reliability of the perception scale. Cronbach’s alpha for the perception scale yielded a value of 0.75, indicating acceptable internal consistency. The study’s data collection was facilitated by eleven research assistants, all of whom were medical practitioners and postgraduate students from various departments within the Faculty of Science. These assistants were trained on effective data collection techniques, which included approaches to obtaining informed consent, explaining the study’s purpose and administering the questionnaire to participants. The research team placed a strong emphasis on ethical data collection practices. Consent was obtained from each participant after they were informed of the study’s objectives and assured of the confidentiality of their responses.

### Data analysis

Data were entered into Microsoft Excel^®^ and analysed using IBM^®^ Statistical Package for the Social Sciences (SPSS) statistical software 20. The analysed data from the questionnaire was presented as frequency tables and percentages. The chi-squared test was used to estimate the association between independent variables and outcome variables (awareness and perception of IFDs). Logistic regression was used to determine factors affecting awareness and perception. A *p*-value ≤ 0.05 was taken for statistical significance at a 95% confidence interval. Awareness of IFDs was determined by the answers to the question ‘Have you ever heard of the following infections?’ with possible answers as aspergillosis, candida infection or candidiasis, mucormycosis, cryptococcosis, histoplasmosis and none of these.

Perception was determined by 10 questions arranged in a 3-point Likert scale in Section C of the questionnaire. A correct response was given 2 points, an uncertain response was given 1 point, while a wrong response was given 0 points each. The respondents’ overall perception was determined by scoring > 50% for good perception and ≤ 50% for poor perception.

### Ethical considerations

Ethical clearance to conduct this study was obtained from the Lagos University Teaching Hospital Health Research Ethics Committee (No. ADM/DSCST/HREC/APP/7069). Informed consent was acquired from every participant before commencement of the interviewer-administered questionnaire. Ethical concepts such as participant confidentiality, voluntary participation and the right to withdraw from the study at any point were fully explained to each participant.

## Results

A total of 1241 members of the general populace in Lagos, Oyo and Cross River states, Nigeria, were involved in this study. The mean age of the participants was 37.1 ± 16.1 years. Just over half of the participants were female (627 [50.5%]). The highest frequency of participants was single (636 [51.2%]), had tertiary education as their highest educational level (538 [43.4%]) and had their household income within the lowest range of less than 30 000.00 naira ($40.00) a month (320 [25.8%]). Over two-thirds of the participants (72.0%) had no chronic diseases, and over three-quarters of the participants (75.3%) were not on any long-term medication. Over half of the participants (644 [51.9%]) had never visited a medical doctor in the past 12 months (Table 1).

**TABLE 1:** Participants’ characteristics and association with awareness of invasive fungal diseases.

Characteristics	Awareness																				
	Overall			IFDs			Aspergillosis			Invasive candidiasis			Cryptococcosis			Mucormycosis			Histoplasmosis		
	Frequency	%	*p*	Frequency	%	*p*	Frequency	%	*p*	Frequency	%	*p*	Frequency	%	*p*	Frequency	%	*p*	Frequency	%	*p*
**Age group (years)**	-	-	-	-	-	0.001*	-	-	0.001*	-	-	0.135	-	-	0.022*	-	-	0.355	-	-	0.045*
< 30	531	42.8	-	128	24.1	-	58	10.9	-	62	11.7	-	43	8.1	-	28	5.3	-	39	7.3	-
30–44	398	32.1	-	71	17.8	-	26	6.5	-	33	8.3	-	19	4.8	-	15	3.8	-	20	5.0	-
45–59	217	17.5	-	26	12.0	-	13	6.0	-	15	6.9	-	14	6.5	-	13	6.0	-	9	4.1	-
≥ 60	95	7.7	-	15	15.8	-	1	1.1	-	11	11.6	-	1	1.1	-	2	2.1	-	1	1.1	-
**Sex**	-	-	-	-	-	0.222	-	-	0.346	-	-	0.044*	-	-	0.906	-	-	0.688	-	-	0.805
Male	614	49.5	-	110	17.9	-	53	8.6		49	8.0	-	39	6.4	-	27	4.4	-	33	5.4	-
Female	627	50.5	-	130	20.7	-	45	7.2	-	72	11.5	-	38	6.1	-	31	4.9	-	36	5.7	-
**Marital status**	-	-	-	-	-	0.010*	-	-	0.002*	-	-	0.494	-	-	0.405	-	-	-	-	-	0.531
Single	636	51.2	-	145	22.8	-	66	10.4	-	70	11.0	-	45	7.1	-	32	5.0	-	40	6.3	-
Married	516	41.6	-	85	16.5	-	31	6.0	-	44	8.5	-	30	5.8	-	24	4.7	-	27	5.2	-
Divorced	31	2.5	-	4	12.9	-	1	3.2	-	3	9.7	-	1	3.2	-	1	3.2	-	1	3.2	-
Widowed	58	4.7	-	6	10.3	-	0	0.0	-	4	6.9	-	1	1.7	-	1	1.7	-	1	1.7	-
**Education**	-	-	-	-	-	0.000*	-	-	0.000*	-	-	0.000*	-	-	0.000*	-	-	0.000*	-	-	0.000*
None	96	7.7	-	0	0.0	-	0	0.0	-	0	0.0	-	0	0.0	-	0	0.0	-	0	0.0	-
Primary	112	9.0	-	1	0.9	-	0	0.0	-	0	0.0	-	1	0.9	-	0	0.0	-	1	0.9	-
Secondary	294	23.7	-	11	3.7	-	3	1.0	-	2	0.7	-	4	1.4	-	1	0.3	-	1	0.3	-
Tertiary	538	43.4	-	159	29.6	-	69	12.8	-	84	15.6	-	49	9.1	-	35	6.5	-	41	7.6	-
Postgraduate	201	16.2	-	69	34.3	-	26	12.9	-	35	17.4	-	23	11.4	-	22	10.9	-	26	12.9	-
**Household income (₦ x1000.00)**	-	-	-	-	-	0.000*	-	-	0.010*	-	-	0.000*	-	-	0.001*	-	-	0.000*	-	-	0.000*
< 30	320	25.8	-	58	18.1	-	21	6.6	-	22	6.9	-	17	5.3	-	9	2.8	-	14	4.4	-
30–50	297	23.9	-	43	14.5	-	15	5.1	-	18	6.1	-	13	4.4	-	9	3.0	-	8	2.7	-
51–100	312	25.1	-	52	16.7	-	23	7.4	-	27	8.7	-	12	3.8	-	9	2.9	-	14	4.5	-
101–300	234	18.9	-	68	29.1	-	29	12.4	-	41	17.5	-	27	11.5	-	24	10.3	-	25	10.7	-
> 300	78	6.3	-	19	24.4	-	10	12.8	-	13	16.7	-	8	10.3	-	7	9.0	-	8	10.3	-
**Doctor visit in past 12 months**	-	-	-	-	-	0.186	-	-	0.798	-	-	0.249	-	-	0.361	-	-	0.044*	-	-	0.209
0	644	51.9	-	117	18.2	-	51	7.9	-	55	8.5	-	37	5.7	-	25	3.9	-	30	4.7	-
1–2	367	29.6	-	76	20.7	-	28	7.6	-	37	10.1	-	22	6.0	-	16	4.4	-	20	5.4	-
3–5	158	12.7	-	27	17.1	-	15	9.5	-	18	11.4	-	10	6.3	-	9	5.7	-	14	8.9	-
≥ 6	72	5.8	-	20	27.8	-	4	5.6	-	11	15.3	-	8	11.1	-	8	11.1	-	5	6.9	-
**Chronic disease(s)**	-	-	-	-	-	0.174	-	-	0.413	-	-	0.000*	-	-	0.066	-	-	0.099	-	-	0.039*
Yes	348	28.0	-	76	21.8	-	31	8.9	-	52	14.9	-	29	8.3	-	22	6.3	-	27	7.8	-
No	893	72.0	-	164	18.4	-	67	7.5	-	69	7.7	-	48	5.4	-	36	4.0	-	42	4.7	-
**Number of long-term medications used**	-	-	-	-	-	0.059	-	-	0.059	-	-	0.859	-	-	0.553	-	-	0.077	-	-	0.006*
0	934	75.3	-	183	19.6	-	80	8.6	-	90	9.6	-	61	6.5	-	41	4.4	-	55	5.9	-
1–2	193	15.6	-	34	17.6	-	11	5.7	-	20	1.4	-	8	4.1	-	9	4.7	-	3	1.6	-
3–5	100	8.1	-	18	18.0	-	4	4.0	-	9	9.0	-	7	7.0	-	5	5.0	-	9	9.0	-
≥ 6	14	1.1	-	5	35.7	-	3	21.4	-	2	14.3	-	1	7.1	-	3	21.4	-	2	14.3	-

**Total**	1241	-	-	240	19.3	-	98	7.9	-	121	9.8	-	77	6.2	-	58	4.7	-	69	5.6	-

Note: Age: 37.1 ± 16.1

IFD, invasive fungal diseases; ₦, naira; *p, p*-value.

* , Statistically significant.

Fewer than one-fifth of the participants (240 [19.3%]) were aware of IFDs (Table 1). Awareness of the various diseases was poor, with the least recognised being mucormycosis (58 [4.7%]), followed by histoplasmosis (69 [5.6%]), cryptococcosis (77 [6.2%]), aspergillosis (98 [7.9%]) and invasive candidiasis (121 [9.8%]) (Table 1). The highest frequency of participants was unsure if they knew someone who had a history of fungal disease (462 [37.2%]), if fungal diseases were not serious (552 [44.5%]), could cause disfigurement (677 [54.6%]) or could cause blindness (66.3%). They were also unsure if fungal diseases could be fatal (625 [50.4%]), could be treated with antibiotics (653 [52.6%]), were difficult to treat (677 [54.6%]), could complicate HIV or diabetes (60.7%) or could be mistaken for tuberculosis (798 [64.3%]). The highest frequency of respondents (509 [41.2%]) reported that they never had a history of fungal disease. Almost two-thirds of participants (820 [66.1%]) had poor perception of IFDs (Table 2).

**TABLE 2:** Perception of invasive fungal diseases amongst participants (*N* = 1241).

Variable	Agree		Not sure		Disagree	
	Frequency	%	Frequency	%	Frequency	%
Personal history of fungal disease	305	24.6	427	34.4	509	41.2
Known person with a history of fungal disease	344	27.7	462	37.2	435	35.1
Fungal diseases are unserious	228	18.4	552	44.5	461	37.2
Fungal diseases can cause disfigurement	380	30.6	677	54.6	184	14.8
Fungal diseases can cause blindness	157	12.7	823	66.3	261	21.0
Some fungal diseases can be fatal	413	33.3	625	50.4	203	16.4
Fungal diseases are treated with antibiotics	395	31.8	653	52.6	193	15.6
Fungal diseases are difficult to treat	249	20.1	677	54.6	315	25.4
Fungal diseases can complicate HIV and diabetes	255	20.6	753	60.7	233	18.8
Fungal diseases can be mistaken for tuberculosis	124	10.0	798	64.3	319	25.7
**Overall perception**						
Good	421	33.9	-	-	-	-
Poor	820	66.1	-	-	-	-

HIV, human immunodeficiency virus.

Higher educational level was associated with greater awareness in the multivariable models for all fungal diseases, as participants with a tertiary education level were much more likely to have awareness of IFDs (adjusted odds ratio [aOR] = 17.082, confidence interval [CI]: 9.269–31.479, *p* < 0.001), aspergillosis (aOR = 19.401, CI: 6.019–62.533, *p* < 0.001), invasive candidiasis (aOR = 41.988, CI: 10.251–171.990, *p* < 0.001), cryptococcosis (aOR = 8.967, CI: 3.488–23.054, *p* < 0.001), mucormycosis (aOR = 34.697, CI: 4.723–254.907, *p* < 0.001) and histoplasmosis (aOR = 16.668, CI: 3.974–69.914, *p* < 0.001) than those with lower levels of education. Female participants were more likely to be aware of invasive candidiasis than male participants (aOR = 1.539, CI: 1.030–2.300, *p* = 0.036), and participants with chronic diseases were twice as likely to have awareness of invasive candidiasis than those with no history of chronic disease (aOR = 2.048, CI: 1.366–3.069, *p* = 0.001). Participants who earned higher within the range of 101 000.00 naira to 300 000.00 naira monthly were over twice more likely to have awareness of histoplasmosis than those earning within the lowest range of less than 30 000.00 naira monthly (aOR = 2.241, CI: 1.037–4.843, *p* = 0.040) (Table 3).

**TABLE 3:** Adjusted odds ratio for study participants’ characteristics associated with awareness of invasive fungal diseases.

Variable	IFDs			Aspergillosis			Invasive Candidiasis			Cryptococcosis			Mucormycosis			Histoplasmosis		
	aOR	95% CI	*p*	aOR	95% CI	*p*	aOR	95% CI	*p*	aOR	95% CI	*p*	aOR	95% CI	*p*	aOR	95% CI	*p*
Age group (years)																		
< 30	1.462	0.760–2.815	0.255	1.085	0.447–2.632	0.857	-	-	-	1.004	0.493–2.045	0.992	-	-	-	2.261	0.933–5.475	0.071
30–44	1.091	0.636–1.873	0.752	0.696	0.329–1.469	0.341	-	-	-	0.541	0.258–1.134	0.104	-	-	-	1.249	0.518–3.011	0.621
45–59	Ref	1.0	-	Ref	1.0	-	-	-	-	Ref	1.0	-	-	-	-	Ref	1.0	-
≥ 60	1.414	0.643–3.108	0.389	0.189	0.023–1.536	0.119	-	-	-	0.140	0.018–1.102	0.062	-	-	-	0.170	0.019–1.492	0.110
**Sex**																		
Male	-	-	-	-	-	-	Ref	1.0	-	-	-	-	-	-	-	-	-	-
Female	-	-	-	-	-	-	1.539	1.030–2.300	0.036*	-	-	-	-	-	-	-	-	-
**Marital status**																		
Single	1.152	0.459–2.894	0.763	3.701	0.437–31.379	0.230	-	-	-	-	-	-	-	-	-	-	-	-
Married	1.095	0.479–2.505	0.829	2.598	0.329–20.552	0.365	-	-	-	-	-	-	-	-	-	-	-	-
Others	Ref	1.0	-	Ref	1.0	-	-	-	-	-	-	-	-	-	-	-	-	-
**Education**																		
Pre-tertiary	Ref	1.0	-	Ref	1.0	-	Ref	1.0	-	Ref	1.0	-	Ref	1.0	-	Ref	1.0	-
Tertiary	17.082	9.269–31.479	0.000*	19.401	6.019–62.533	0.000*	41.988	10.251–171.990	0.000*	8.967	3.488–23.054	0.000*	34.697	4.723–254.907	0.000*	16.668	3.974–69.914	0.000*
**Household income (₦ x1000)**																		
< 30	Ref	1.0	-	Ref	1.0	-	Ref	1.0	-	Ref	1.0	-	Ref	1.0	-	Ref	1.0	-
30–50	0.756	0.470–1.218	0.251	0.840	0.413–1.711	0.631	0.871	0.443–1.711	0.689	0.894	0.417–1.914	0.773	1.062	0.409–2.762	0.901	0.733	0.294–1.828	0.505
51–100	0.767	0.476–1.234	0.274	1.188	0.608–2.324	0.614	1.045	0.564–1.934	0.889	0.674	0.301–1.508	0.337	0.775	0.299–2.010	0.600	1.193	0.524–2.714	0.674
101–300	1.110	0.684–1.800	0.673	1.605	0.818–3.148	0.169	1.597	0.896–2.845	0.112	1.685	0.825–3.445	0.152	2.011	0.895–4.518	0.091	2.241	1.037–4.843	0.040*
> 300	0.732	0.380–1.412	0.352	1.603	0.669–3.842	0.290	1.209	0.563–2.596	0.627	1.432	0.550–3.730	0.462	1.513	0.528–4.334	0.440	2142	0.789–5.816	0.135
**Doctor visit in past 12 months**																		
0	-	-	-	-	-	-	-	-	-	-	-	-	Ref	1.0	-	-	-	-
1–2	-	-	-	-	-	-	-	-	-	-	-	-	0.826	0.429–1.590	0.567	-	-	-
3–5	-	-	-	-	-	-	-	-	-	-	-	-	1.052	0.470–2.358	0.902	-	-	-
≥ 6	-	-	-	-	-	-	-	-	-	-	-	-	2.200	0.908–5.327	0.081	-	-	-
**Chronic disease(s)**																		
Yes	-	-	-	-	-	-	2.048	1.366–3.069	0.001*	-	-	-	-	-	-	2.228	1.249–3.976	0.007*
No	-	-	-	-	-	-	Ref	1.0	-	-	-	-	-	-	-	Ref	1.0	-
**Number of long-term medications used**																		
0	-	-	-	-	-	-	-	-	-	-	-	-	-	-	-	4.096	1.198–14.001	0.025*
1–2	-	-	-	-	-	-	-	-	-	-	-	-	-	-	-	Ref	1.0	-
3–5	-	-	-	-	-	-	-	-	-	-	-	-	-	-	-	8.178	2.042–32.749	0.003*
≥ 6	-	-	-	-	-	-	-	-	-	-	-	-	-	-	-	8.170	1.130–59.074	0.037*

Note: Others included divorced or widowed individuals. Pre-tertiary, no formal education, primary or secondary.

Ref, reference; ₦, naira; aOR, adjusted odds ration; CI, confidence interval.

* , Statistically significant.

Participants with chronic diseases were twice as likely to have awareness of histoplasmosis than those with no history of chronic disease (aOR = 2.228, CI: 1.249–3.976, *p* = 0.007). There was a convex relationship between the participants’ history of long-term medications being used and their awareness of histoplasmosis, as those who were not on any long-term medications (aOR = 4.096, CI: 1.198–14.001, *p* = 0.025), those who were on three to five medications (aOR = 8.178, CI: 2.042–32.749, *p* = 0.003) and those on six or more medications (aOR = 8.170, CI: 1.130–59.074, *p* = 0.037) were more likely to be aware of histoplasmosis compared with those who were on one to two medications (Table 3).

Higher educational level was also associated with better perception of fungal diseases, as participants with secondary (aOR = 2.116, CI: 1.005–4.454, *p* = 0.049), tertiary (aOR = 5.420, CI: 2.615–11.234, *p* < 0.001) and postgraduate education (aOR = 5.372, CI: 2.485–11.616, *p* < 0.001) as their highest educational level were more likely to have good perception compared with those with no formal education. Participants who were single (aOR = 6.772, CI: 1.891–24.252, *p* = 0.003), married (aOR = 4.947, CI: 1.435–17.052, *p* = 0.011) and widowed (aOR = 4.384, CI: 1.032–18.627, *p* = 0.045) were more likely to have good perception compared with those who were divorced. Participants who had visited the doctor three to five times in the past 12 months were about one and a half times more likely to have a good perception of fungal diseases (aOR = 1.485, CI: 1.009–2.185, *p* = 0.045) compared with those who never visited a doctor in the past 12 months (Table 4).

**TABLE 4:** Relationship between study participants’ characteristics and perception of invasive fungal diseases.

Variable	Perception				*χ* ^2^	*p* (Fishers’ exact)	aOR	95% CI	*p*
	Good (*n* = 421)		Poor (*n* = 820)						
	Frequency	%	Frequency	%					
**Age group (years)**	-	-	-	-	8.978	0.030*	-	-	-
< 30	196	36.9	335	63.1	-	-	1.175	0.570–2.422	0.661
30–44	137	34.4	261	65.6	-	-	1.319	0.687–2.535	0.405
45–59	67	30.9	150	69.1	-	-	1.605	0.831–3.100	0.159
≥ 60	21	22.1	74	77.9	-	-	Reference	1.0	-
**Sex**	-	-	-	-	0.990	0.320	-	-	-
Male	200	32.6	414	67.4	-	-	-	-	-
Female	221	35.3	406	64.7	-	-	-	-	-
**Marital status**	-	-	-	-	19.437	0.000*	-	-	-
Single	239	37.6	397	62.4	-	-	6.772	1.891–24.252	0.003*
Married	169	32.8	347	67.2	-	-	4.947	1.435–17.052	0.011*
Divorced	3	9.7	28	90.3	-	-	Reference	1.0	-
Widowed	10	17.2	48	82.8	-	-	4.384	1.032–18.627	0.045*
**Education**	-	-	-	-	100.282	0.000*	-	-	-
None	10	10.4	86	89.6	-	-	Reference	1.0	-
Primary	15	13.4	97	86.6	-	-	1.271	0.536–3.014	0.586
Secondary	66	22.5	228	77.5	-	-	2.116	1.005–4.454	0.049*
Tertiary	237	44.1	301	55.9	-	-	5.420	2.615–11.234	0.000*
PG	93	46.3	108	53.7	-	-	5.372	2.485–11.616	0.000*
**Household income (₦ x1000)**	-	-	-	-	30.780	0.000*	-	-	-
< 30	91	28.4	229	71.6	-	-	Reference	1.0	-
30–50	86	29.0	21	71.0	-	-	1.028	0.707–1.495	0.884
51–100	100	32.1	212	67.9	-	-	1.009	0.687–1.482	0.963
101–300	103	44.0	131	56.0	-	-	1.334	0.879–2.025	0.175
> 300	41	52.6	37	47.4	-	-	1.671	0.943–2.962	0.078
**Doctor visit in past 12 months**	-	-	-	-	13.795	0.003*	-	-	-
0	189	29.3	455	70.7	-	-	Reference	1.0	-
1–2	139	37.9	228	62.1	-	-	1.190	0.892–1.588	0.237
3–5	67	42.4	91	57.6	-	-	1.485	1.009–2.185	0.045*
> 5	26	36.1	46	63.9	-	-	1.179	0.675–2.057	0.563
**Chronic disease(s)**	-	-	-	-	1.761	0.205	-	-	-
Yes	128	36.8	220	63.2	-	-	-	-	-
No	293	32.8	600	67.2	-	-	-	-	-
**Number of medications used for chronic diseases**	-	-	-	-	0.409	0.939	-	-	-
0	321	34.4	613	65.6	-	-	-	-	-
1–2	63	32.6	130	67.4	-	-	-	-	-
3–5	32	32.0	68	68.0	-	-	-	-	-
≥ 6	5	35.7	9	64.3	-	-	-	-	-

aOR, adjusted odds ration; CI, confidence interval; PG, postgraduate; ₦, naira; *p, p*-value.

* , Statistically significant.

## Discussion

Serious fungal infections are associated with high morbidity, mortality and cost of care.^2^ Community awareness of invasive fungal infections was low, with only less than a fifth of the participants in this study having awareness of any one or more of the IFDs. This is despite the high burden of infection in this population, where it has been estimated that up to 12% of the population suffer from a serious fungal infection annually.^9^ Our finding in the general populace is likely a reflection of the general low level of awareness of fungal infections in our setting. An earlier study by Oladele et al. had demonstrated such low levels even among medical personnel in the same country.^16^

Invasive candidiasis, which was the IFD with the highest level of awareness (9.8%), still had less than a tenth of the participants reporting their awareness of it. This pattern of awareness is similar to a recent study on awareness of fungal diseases in America, where public awareness was seen to be low. In that study, the IFD with the highest level of public awareness was similarly candidiasis (24.6%).^3^ It was purported that the relatively higher awareness of candidiasis compared with other fungal infections was a result of its frequent involvement in vulvovaginal infections, which is a common infection causing millions of outpatient visits nationwide.^1,3^ In our study, perception of fungal disease was also low, with about two-thirds (820 (66.1%)) having poor perception. This poor awareness and perception of serious fungal infections may have a negative effect on the health-seeking behaviour of infected individuals in the country. Those unaware of these conditions may not access medical care early enough, allowing such diseases to thrive, thus increasing the burden of serious fungal infections in the populace. Our study revealed that higher educational level was associated with greater awareness for all fungal diseases and associated with good perception of fungal diseases. Furthermore, those with frequent doctor visits demonstrated higher awareness levels. Participants who had visited a doctor six or more times in the past year had the highest awareness of IFDs (27.8%) compared to those who had not visited a doctor (18.2%). This suggests that healthcare encounters play a crucial role in increasing awareness of fungal diseases, emphasising the importance of patient education in clinical settings.

Factors such as occupation and endemicity have been suggested to influence awareness of serious fungal infections^3^ and education somewhat influences occupational class and knowledge of endemic diseases. These might explain the observed higher awareness and perception in the educated population. However, this associative explanation is limited because other factors such as experience of any of the disease(s), knowledge of an infected person(s) or health literacy, which are difficult to measure, may be responsible for our findings.

Despite the widespread dearth of awareness and the critical need to improve awareness, there is a scarcity of literature assessing the awareness and perception of fungal infections in various populations. Such data are important for needs assessment and planning of intervention to ensure that there is adequate awareness and early diagnosis and treatment. There need to be concerted efforts to improve public awareness and knowledge in order to prevent and control IFDs. This brings to the fore the role of international days set aside for awareness creation. One such initiative is the Fungal Disease Awareness Week, which emphasises early detection and life-saving treatment of serious fungal infections.^17^ Another is the World Aspergillosis Day, which raises awareness of aspergillosis, a fungal infection with varying manifestations.^18^ It is quite commendable that the most recent World Aspergillosis Day was dedicated to raising the awareness and knowledge and highlighting the experiences of the general population living with this infection.^19^

In recent years, various initiatives have emerged globally to promote awareness of fungal diseases. Notably, African Fungus Day has helped to highlight the significance of fungal infections across the continent through regional collaboration and public outreach campaigns.^20^ Additionally, the Global Action for Fungal Infections (GAFFI) initiative, Fight Fungal Infections, continues to support awareness and advocacy efforts worldwide.^21^ Educational programmes like ‘Fungi Connect’, targeted at children and young people, have also played a role in fostering early understanding and interest in fungal diseases through engaging learning tools and activities.^22^

Awareness creation in LMICs such as Nigeria still has a long way to go in spite of these laudable measures. A major obstacle to raising awareness and reducing the burden of fungal infections is lack of funding. Fungal infections are grossly neglected and ranked low on the priority for funding by most governments and prospective donors even though there is a growing population of susceptible individuals and a threat to public health posed by these diseases.^23^ It is expected that advocacy efforts such as those of medical mycology societies will result in improvement in funding opportunities for fungal infection awareness, diagnosis and management.^24^

The role of mass media, particularly digital platforms, in shaping health perceptions cannot be overstated. In recent years, social media has proven to be a powerful tool in promoting awareness about various infectious diseases, from Ebola to coronavirus disease 2019 (COVID-19).^14^ Leveraging these platforms to educate the public on fungal infections could bridge the awareness gap, foster early recognition of symptoms and encourage timely medical intervention. Community outreach programmes, integration of fungal disease education into primary healthcare systems and multilingual health promotion campaigns can also play a significant role in reaching diverse populations.^16^

Collaboration with community leaders, healthcare providers and local media outlets could enhance outreach efforts and ensure that accurate, accessible information reaches a broad audience. Furthermore, integrating IFD awareness programmes into school curricula, particularly in secondary and tertiary institutions, may foster greater understanding and encourage preventive practices from younger ages. The findings from this survey will also serve as a baseline for comparison with future surveys.

## Conclusion

Awareness and perception of IFDs in Nigeria are low. The low level of awareness and perception of fungal infections requires determined efforts through education, advocacy and funding to ensure fungal infections are identified early and adequately controlled. This study highlights the critical need for increased public health awareness and education on IFDs in Nigeria. Recognising this crucial gap in awareness and perception of IFDs, the Fungal Diseases Surveillance Program Committee has initiated a targeted social media campaign (#ThinkFungal). This campaign is strategically designed to first engage clinicians – who play a pivotal role in early detection and management – and subsequently reach the general public, focusing on the promotion of early hospital presentation at the onset of symptoms to improve overall outcomes. By leveraging the wide reach of digital platforms, the campaign aims to improve awareness, promote timely diagnosis and ultimately reduce the morbidity and mortality associated with these often-overlooked infections.

### Limitations

This study has limitations that should be acknowledged. Firstly, although it provides valuable insights into the awareness and perception of IFDs among Nigerians, the study was conducted in only three states – Lagos, Oyo and Cross River – which may limit the generalisability of the findings to the entire country. Regional differences in education, healthcare access and cultural practices across Nigeria may influence awareness levels, and caution should be exercised when extrapolating the results nationally. Secondly, data collection relied on self-reported responses, which may be subject to recall or social desirability bias. Additionally, while efforts were made to reach a diverse cross section of the population through stratified sampling, some demographic subgroups may have been under-represented. Despite these limitations, the study offers a useful foundation for larger, nationally representative research on fungal disease awareness in Nigeria.

## Appendix 1

### Questionnaire

#### Awareness and perception of fungal diseases amongst Nigeria’s general populace

Please assist in filling this questionnaire as honestly as you can. Participation is voluntary but would be greatly appreciated.

##### Section A: Biodata of do the healthcare

1. Age (years):    □ 18-29    □ 30-44    □ 45-59    □ 60 and above
2. State/City of residence: ______________
3. Sex:    □ Male    □ Female
4. Marital status:    □ Single    □ Married    □ Divorced    □ Widowed
5. Educational level:    □ None    □ Primary    □ Secondary    □ Tertiary    □ Postgraduate
6. Occupation: ______________
7. Household monthly income in naira:
  □ Less than 30,000    □ 30,000 – 50,000    □ 51,000 – 100,000    □ 101,000 – 300,000    □ Above 300,000
8. Have you/ Are you having any of the conditions below
  □ Allergy    □ Hypertension    □ Diabetes    □ Cancer    □ Chronic lung disease    □ Chronic Liver Disease (Hepatitis)    □ None    □ Others (please specify) ______________
9. How often have you seen a doctor in the last 12 months?
  □ 1 -2 times    □ 3 – 5 times    □ More than 5 times
10. Are you currently taking drug(s) for any disease?    □ Yes    □ No
11. If yes, was it prescribed by a doctor?    □ Yes    □ No
12. If yes, how many drugs? ______________

##### Section B: Awareness of what

13 Have you heard of Invasive Fungal Infections?    □ Yes    □ No
14 Have you heard of the following infections? *Select all that apply*
  □ Candidiasis (Invasive)    □ Cryptococcosis    □ Aspergillosis    □ Mucormycosis    □ Histoplasmosis    □ None of these

##### Section C: Perception of what?

**Table:**

Number	Statement	Agree	Not sure	Disagree
1.	I have had a fungal disease (life-threatening)			
2.	I have known someone who had a fungal disease (life-threatening)			
3.	Fungal diseases are not serious			
4.	Fungal diseases can cause disfigurement			
5.	Fungal disease can cause blindness			
6.	Fungal diseases can be fatal			
7.	Fungal diseases are treated with antibiotics			
8.	Fungal diseases are difficult to treat			
9.	Fungal diseases complicate known diseases like HIV and diabetes			
10.	Fungal diseases can be mistaken for tuberculosis			
